# Increased risk of cardiovascular disease among kidney cancer survivors: a nationwide population-based cohort study

**DOI:** 10.3389/fonc.2024.1420333

**Published:** 2024-07-12

**Authors:** Minji Jung, Eunjung Choo, Shufeng Li, Zhengyi Deng, Jinhui Li, Mingyi Li, Satvir Basran, Sukhyang Lee, Marvin E. Langston, Benjamin I. Chung

**Affiliations:** ^1^ Department of Urology, Stanford University Medical Center, Stanford, CA, United States; ^2^ Department of Clinical Pharmacy, School of Pharmacy, Ajou University, Suwon, Republic of Korea; ^3^ Department of Dermatology, Stanford University Medical Center, Stanford, CA, United States; ^4^ Department of Epidemiology and Population Health, School of Medicine, Stanford University, Stanford, CA, United States

**Keywords:** kidney cancer, cardiovascular disease, cancer survivorship, primary prevention, cardiooncology

## Abstract

**Background:**

Cardiovascular disease (CVD) is a major concern of morbidity and mortality among cancer survivors. However, few evidence exists on the short- and long-term risk of CVD in kidney cancer (KCa) survivors.

**Methods:**

In this nationwide, large population-based retrospective cohort study, we used the Korean national health insurance and medical checkup survey linkage database (2007-2021), drawn from the entire Korean population. We included adults diagnosed with KCa as the first primary cancer and matched them to an individual without KCa at a 1:5 ratio. The primary outcome was CVD incidence, including myocardial infarction, stroke, atrial fibrillation, heart failure, peripheral arterial occlusion, and venous thromboembolism (VTE). We evaluated CVD risk at 6 months, 1 year, and 5 years following cancer diagnosis, using Fine-Gray competing risk models that accounted for death as a competing factor.

**Results:**

A total of 149,232 participants were included (KCa survivors: N=20,093 and matched non-KCa individuals: N=129,139). After 6-month follow-up, KCa survivors showed an increased risk of CVD compared to the general population (subdistribution hazard ratio (HR) 2.70, 95% confidence interval (CI) 2.31-3.15). After 1 year, KCa survivors had a higher risk of CVD (HR=1.77, 95% CI: 1.56-2.00). After 5 years, this elevated CVD risk remained (HR=1.10, 95% CI: 1.03-1.18), with VTE identified as the primary contributing disease (HR=3.05, 95% CI:2.59-3.59).

**Conclusion:**

KCa survivors had an increased risk of CVD up to 5 years after cancer diagnosis compared to the general population. Our findings emphasize the importance of comprehensive healthcare management for both CVD and KCa throughout cancer survivorship.

## Introduction

Kidney cancer (KCa) survival has been rising for decades (1, 2). The current 5-year relative survival for all cases of KCa is 78.0%, and it increases to 93.2% for localized KCa, which accounts for approximately two-thirds of all cases (1). The high survival is due in large part to the significant improvements in cancer treatments (3) and the increasing frequency of abdominal imaging on non-specific diverse conditions such as gastrointestinal diseases, which may lead to early detection (4).

In this growing population of KCa survivors with a longer life expectancy, cardiovascular disease (CVD) is the leading cause of morbidity and non-cancer mortality. Among all KCa patients, CVD contributes nearly 17% of all-cause mortality (4). This percentage increases to 25% in patients with early-stage disease (5), and 42% in those aged over 65 years with early-stage disease (6). This could be attributable to common pathophysiological mechanisms (e.g., oxidative stress, chronic inflammation, and prothrombotic state), shared risk factors (e.g., hypertension, smoking, and obesity) (7), or cardiotoxicity associated with systematic treatments or radiation therapy (8–10). With an aging population (11), the burden for CVD among KCa survivors is expected to increase. However, KCa survivors receive less attention regarding their CVD risk than survivors from other cancer sites, such as breast cancer or lymphoma (12–16). This is likely due to the fact that the vast majority, nearly 80%, of KCa patients receive nephrectomy (4), whereas the current CVD management guidelines in cancer patients predominantly focus on cardiotoxic adverse effects associated with cancer systematic treatments, such as chemotherapy, targeted therapy, or radiation therapy (8, 9, 17, 18).

To date, few studies have investigated whether, and if so, to what extent and for how long, KCa survivors face an elevated CVD risk compared to the general population. Understanding CVD risk in KCa survivors would be the foundational step to guide the prevention and management of CVD, while simultaneously providing optimal cancer care, and ultimately reduce their morbidity and mortality. Therefore, our study aimed to investigate the short- and long-term risk of CVD in KCa survivors compared to the general population.

## Method

### Data source

The Republic of Korea implements the National Health Insurance Service (NHIS) program, which is the single-payer, national health insurance program. The NHIS program covers over 97% of the entire Korean population, which is approximately 50 million people. The Korean NHIS provides the population-based health insurance data, which was linked to the national medical checkup survey data (19). We used the Korean national health insurance and medical checkup survey linkage data from 2007 to 2021, which was de-identified, and this enables us to conduct the study on a nationwide scale. The data includes the following information: 1) patient information including age, sex, income level; 2) diagnosis and procedure information with clinic and hospital visit records; 3) prescription information; 4) vital status and date of death; and 5) physical examination measures (e.g., body mass index (BMI)) and lifestyle factors (e.g., smoking status, alcohol drinking, and physical activity). Information of diagnoses was recorded according to the International Classification of Diseases, 10th Revision (ICD-10). This study was approved by the Institutional Review Board of Ajou University (IRB no.202209-HB-EX-002).

### Study population

In this nationwide population-based retrospective cohort study, we included adults (≥18 years) who were diagnosed with KCa as their first primary cancer during a cohort entry period (2010-2020), drawn from the entire Korean population. The KCa diagnosis was identified using ICD-10 code (C64), which was supported by V code (V193 and V194). V code is issued by the NHIS, allowing the confirmation of cancer diagnostic codes based on clinicopathologic assessments (20). A comparison group was also generated from the entire Korean population. This comparison group comprised individuals without a diagnosis of KCa during the study period and was matched to KCa patients based on sex and age at a 1:10 ratio using frequency matching methods. Further, to determine the index date for the comparison group, we matched them by using individual matching methods according to sex and age in the same year with an individual with KCa at a 1:5 ratio. The index date for individuals with KCa was defined as the first diagnosed date of KCa. The index date for those without KCa was assigned the same date as their matched KCa patient.

**Figure 1:**
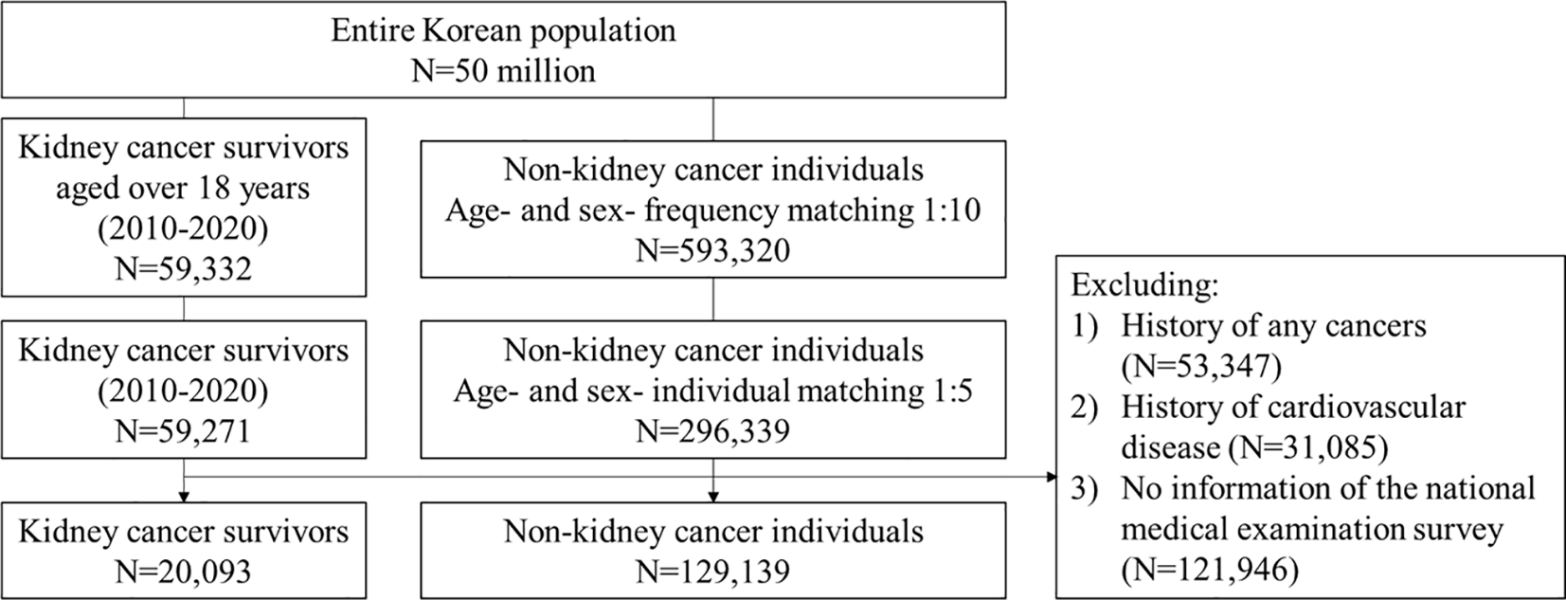
Flowchart. We included adults with a primary KCa and age- and sex-matched non-KCa individuals. CVD, cardiovascular disease; KCa, kidney cancer.

We excluded individuals as follows: 1) those aged under 18 years at the index date; 2) to ascertain the first primary KCa case, individuals with a history of any cancers, except for non-melanoma skin cancer, prior to the index date; 3) to capture incident CVD events, individuals with a history of myocardial infarction, ischemic stroke/transient ischemic attack, hemorrhagic stroke, atrial fibrillation (AF), heart failure, peripheral arterial occlusion (PAO), and venous thromboembolism (VTE) prior to the index date (15, 21, 22); and 4) to conduct a complete case analysis, those had no information of medical checkup survey data within two years before the index date (**Figure 1** and **Supplementary Figure S1**). According to the National Cancer Institute, we defined individuals diagnosed with KCa from the time of cancer diagnosis throughout their life as “KCa survivors” (23). We referred to the matched individuals in the comparison group as “non-KCa individuals”.

### Study outcomes

The primary outcome was an incident CVD, a composite of myocardial infarction, ischemic stroke/transient ischemic attack, hemorrhagic stroke, AF, heart failure, PAO, and VTE (15, 21, 22). Incident myocardial infarction, ischemic stroke/transient ischemic attack, hemorrhagic stroke, and heart failure were defined as a hospitalization or emergency department visit with a primary diagnostic code corresponding to ICD-10 codes (24–26). Incident AF, PAO, and VTE were defined as a hospitalization or emergency department with a primary diagnostic code or at least two separate records of outpatient visits with a primary diagnostic code (21, 22). The secondary outcomes were individual components of CVD and all-cause mortality. Detailed information is provided in **Supplementary Table S1**. To assess their short- and long-term risk, we set three time points in the main analysis: 6 months, 1 year, and 5 years after the index date (21, 22). Additionally, we assessed CVD risk at different time points: 3 months, 2 years, 3 years, and 4 years following cancer diagnosis. Each participant was followed from the index date to the occurrence of outcome, death, or end of the study follow-up (i.e., each time point or December 31, 2021), whichever came first.

### Potential confounders

We identified potential confounders including sociodemographic variables, comorbidities, medications, and lifestyle factors (2, 3, 7, 27, 28). Sociodemographic variables included sex, age, income level, and number of visits to medical institutions. Comorbidities included hypertension, dyslipidemia, diabetes mellitus with or without complications, chronic obstructive pulmonary disease (COPD), chronic kidney cancer (CKD, which included stage 1-4), end-stage renal disease (ESRD), mild to moderate and severe liver disease, and peripheral vascular disease (PVD) (**Supplementary Table S1**). Charlson Comorbidity Index (CCI) was calculated to estimate an individual’s medical burden of disease (29). Medications included angiotensin converting enzyme inhibitors, angiotensin receptor blockers, beta-blockers, calcium channel blockers, diuretics, statin, and metformin. Lifestyle factors included BMI, smoking, drinking, and physical activity.

Age was categorized into three groups: 18-50, 50-65, and over 65 years. Income level was divided into three equal parts. Patients who visited medical institutions more than 24 times within one year prior to the index date were defined as “frequent visitors”. BMI was categorized into three groups: <23.0 kg/m^2^ (underweight/normal weight), 23.0-25.0 kg/m^2^ (overweight), and ≥ 25.0 kg/m^2^ (obese) (30). Smoking was classified into three groups: never smokers, former smokers, and current smokers (30). Alcohol drinking was classified into three groups: non-drinkers, moderate drinkers (1-4 days/week), and heavy drinkers (≥ 5 days/week) and physical activity was divided into three groups: non-active, moderately active (1-4 days/week), and active (≥ 5 days/week) (30).

### Statistical analysis

We used the Chi-square test for categorical variables and paired *t* test or Wilcoxon’s rank-sum test for continuous variables. We calculated cumulative incidence and incidence rate (IR) per 1,000 person-years following the index date. We conducted the Fine-Gray competing risk models to compare the risk of CVD between the study groups with accounting for a competing risk of death. The models were adjusted for the potential confounders listed above. We used the Cox proportional hazards regression model for all-cause mortality and adjusted it in the same way as the Fine-Gray model.

To assess the differential effects on CVD risk according to various patient characteristics, we performed several subgroup analyses as follows: age (<50, 50-65, and ≥65 years), sex, index year (2010-2014 and 2015-2020), comorbidities (hypertension, dyslipidemia, diabetes mellitus, COPD, CKD, ESRD, liver disease, and PVD) and lifestyle factors (BMI, smoking, drinking, and physical activity). To identify significant differences within subgroups, an interaction test between KCa and a subgroup was performed. Moreover, four sensitivity analyses were conducted. First, we assumed that KCa survivors who lived longer compared to those who lived shorter are more likely to be at the earlier stage or less susceptible to severe disease, which may lead to different risks of CVD (1, 4). To assess this assumption, we redefined KCa survivors as individuals who lived longer than 2 years after cancer diagnosis and repeated the main analysis. Furthermore, we reapplied a time frame of 5 years. Second, to consider the relationship of older age with a higher risk of CVD (31), we restricted our study participants who were aged over 40 years. Third, to assess the robustness of our adjusted model, we reconducted the analysis by creating a pseudo study cohort using the inverse probability of treatment weighting method. Lastly, in order to investigate whether including only individuals, who participated in the medical checkup survey, introduced any selection bias, we incorporated both participants of the medical checkup survey and non-participants, collectively termed as the “total population”. We set up statistical significance as a two-tailed p value < 0.05. All data analyses were conducted using SAS Version 9.4 and R Version 4.0.

## Results

### Baseline characteristics

A total of 149,232 participants were included: 20,093 were KCa survivors and 129,139 were non-KCa individuals (**Figure 1**). Overall, the median (interquartile range, IQR) age was 59 (50-67) years at the index date and approximately 28% were women. The most prevalent comorbidity was hypertension (36.9%), followed by dyslipidemia (34.2%), mild to moderate liver disease (24.7%), and diabetes with or without complications (23.0%). The median (IQR) BMI was 24.2 (22.2-26.2) kg/m^2^. Approximately 25.5% were current smokers; 6.7% were heavy drinkers; and 24.2% were physically active. The overall median (IQR) follow-up time (years) was 5.48 (3.32-8.17). KCa survivors were more likely to be older and to have more comorbidities compared with non-KCa individuals (**Table 1** and **Supplementary Table S2**). Baseline characteristics of the total population are presented in **Supplementary Table S3**.

**Table 1:** Baseline characteristics of study population.

	Overall		KCa survivors		Non-KCa individuals	
	N=149,232		N=20,093		N=129,139	
	N	(%)	N	(%)	N	(%)
Sex						
Male	107,117	71.78	14,562	72.47	92,555	71.67
Female	42,115	28.22	5,531	27.53	36,584	28.33
Age (Median (IQR))	59.00	(50-67)	57.00	(48-65)	59.00	(50-67)
Under 50 years	35,885	24.05	5,628	28.01	30,257	23.43
50-65 years	65,976	44.21	9,173	45.65	56,803	43.99
Over 65 years	47,371	31.74	5,292	26.34	42,079	32.58
Income level^a^						
Low	31,256	20.94	3,768	18.75	27,488	21.29
Middle	41,363	27.72	5,350	26.63	36,013	27.89
High	73,571	49.30	10,601	52.76	62,970	48.76
Missing	3,042	2.04	374	1.86	2,668	2.07
Number of visits to medical institutions^b^ (Median (IQR))	13.00	(6-24)	17.00	(10-28)	13.00	(6-23)
Frequent visitors	37,951	25.43	6,656	33.13	31,295	24.23
Comorbidity						
Hypertension	55,009	36.86	9,617	47.86	45,392	35.15
Dyslipidemia	50,962	34.15	9,295	46.26	41,667	32.27
Diabetes without complications	26,018	17.43	4,981	24.79	21,037	16.29
Diabetes with complications	8,265	5.54	1,648	8.20	6,617	5.12
COPD	11,941	8.00	2,403	11.96	9,538	7.39
Chronic kidney disease	1,396	0.94	568	2.83	828	0.64
End-stage renal disease	797	0.53	360	1.79	437	0.34
Mild to moderate liver disease	36,867	24.70	8,425	41.93	28,442	22.02
Severe liver disease	396	0.27	82	0.41	314	0.24
Peripheral vascular disease	15,731	10.54	2,372	11.81	13,359	10.34
CCI scores^c^ (Median (IQR))	1.00	(0-2)	1.00	(1-2)	1.00	(0-2)
0	58,897	39.47	4,915	24.46	53,982	41.80
1	42,946	28.78	5,830	29.02	37,116	28.74
2	24,674	16.53	4,376	21.78	20,298	15.72
Over 3	22,715	15.22	4,972	24.74	17,743	13.74
Medication						
ACEI/ARB	35,417	23.73	6,351	31.61	29,066	22.51
Beta-blockers	9,019	6.04	1,601	7.97	7,418	5.74
Calcium channel blockers	32,493	21.77	5,644	28.09	26,849	20.79
Diuretics	16,708	11.20	2,990	14.88	13,718	10.62
Statin	27,809	18.63	4,401	21.90	23,408	18.13
Metformin	15,410	10.33	2,394	11.91	13,016	10.08
BMI (Median (IQR))	24.20	(22.2-26.2)	24.70	(22.7-26.8)	24.10	(22.2-26.1)
<23 kg/m2 (Normal weight)	50,062	33.55	5,562	27.68	44,500	34.46
<25 kg/m2 (Overweight)	39,984	26.79	5,114	25.45	34,870	27.00
>=25 kg/m2 (Obese)	59,186	39.66	9,417	46.87	49,769	38.54
Smoking						
None	73,382	49.17	9,450	47.03	63,932	49.51
Former smoker	37,788	25.32	5,137	25.57	32,651	25.28
Current smoker	38,062	25.51	5,506	27.40	32,556	25.21
Drinking						
None	70,846	47.47	9,264	46.11	61,582	47.69
Moderate (1-4 days/week)	68,432	45.86	9,560	47.58	58,872	45.59
Heavy (5+ days/week)	9,954	6.67	1,269	6.32	8,685	6.73
Physical activity						
None	65,083	43.61	8,609	42.85	56,474	43.73
Moderate (1-4 days/week)	48,062	32.21	6,684	33.27	41,378	32.04
Active (5+ days/week)	36,087	24.18	4,800	23.89	31,287	24.23

ACEI/ARB, angiotensin converting enzyme inhibitors/angiotensin receptor blocker; CCI, Charlson Comorbidity Index; COPD, chronic obstructive pulmonary disease; IQR, interquartile range; KCa, kidney cancer; SMD, absolute standardized mean difference.

^a^Income level was identified based on the third quartile.

^b^Patients who visited medical institutions more than 24 times during one year prior to the index date were defined as “frequent visitors”.

^c^In the classification process of Charlson Comorbidity Index, we excluded two comorbidity groups, namely malignancy and metastatic carcinoma, as they could be our study exposure, a kidney cancer itself.

### Primary outcome

**Figure 2:**
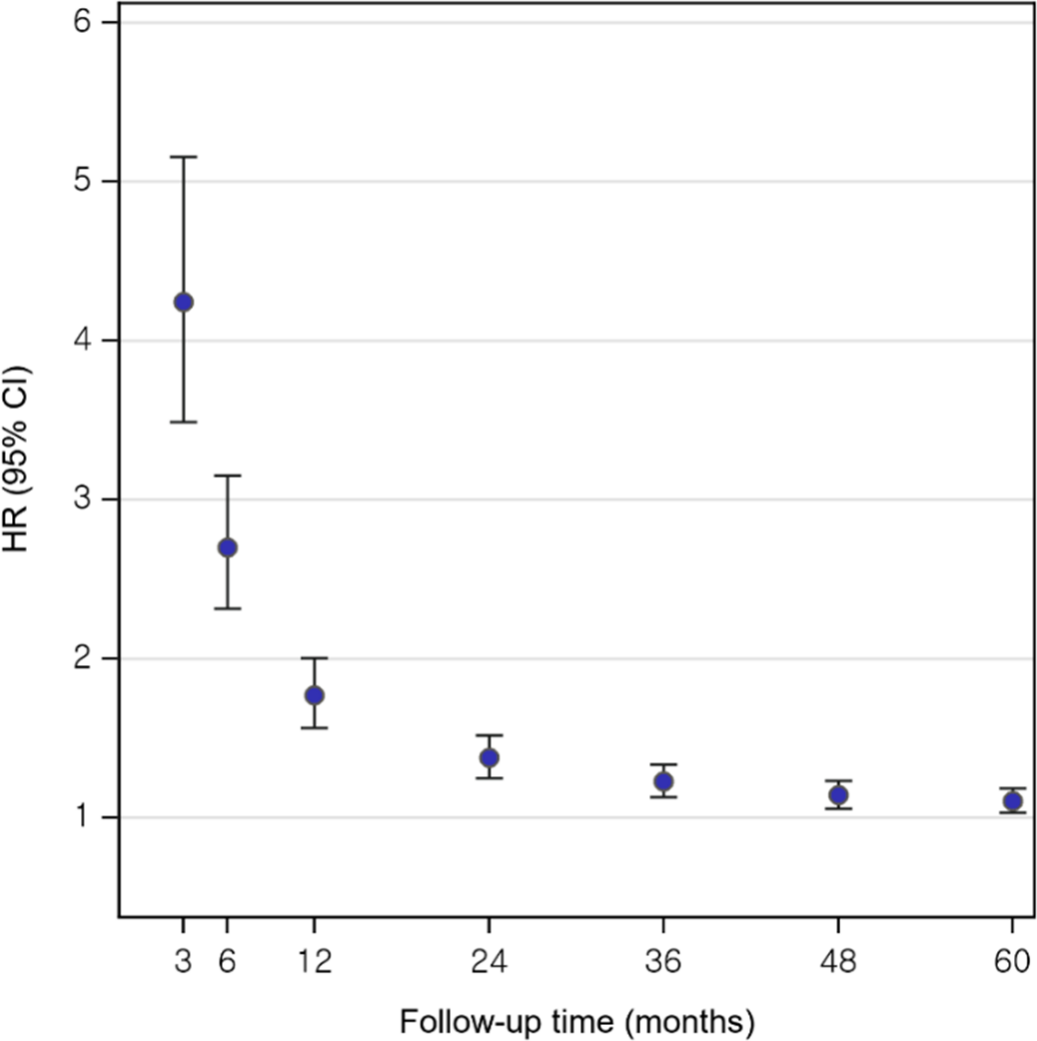
Increased risk of cardiovascular disease among kidney cancer survivors compared to the general population. Compared to non-KCa individuals, increased risks of CVD were shown in KCa survivors up to 5-years after a cancer diagnosis. CI, confidence interval; CVD, cardiovascular disease; HR, hazard ratio; KCa, kidney cancer.

The risk of CVD among KCa survivors was increased compared to the general population (**Table 2** and **Figure 2**). At 6 months after cancer diagnosis, 264 (1.31%) CVD events occurred in KCa survivors (IR=26.74 per 1,000 person-years), while 600 (0.46%) events occurred in non-KCa individuals (IR=9.32 per 1,000 person-years). Compared to non-KCa individuals, KCa survivors showed a 2.7-times higher risk of CVD (adjusted subdistribution hazard ratio (HR) 2.70, 95% confidence interval (CI) 2.31-3.15). At 1 year following the index date, 358 (1.78%) and 1,236 (0.96%) CVD events occurred in KCa survivors (IR=18.36 per 1,000 person-years) and in non-KCa individuals (IR=9.64 per 1,000 person-years), respectively. KCa survivors had a 1.8-fold increased risk of CVD compared to the comparison group (HR=1.77, 95% CI: 1.56-2.00). After 5 years, 993 (4.94%) CVD events occurred in KCa survivors (IR=10.81 per 1,000 person-years), while 5,578 (4.32%) events occurred in non-KCa individuals (IR=8.97 per 1,000 person-years). The elevated risk of CVD remained after 5 years post cancer diagnosis (HR=1.10, 95% CI: 1.03-1.18).

**Table 2:** Risks of cardiovascular diseases and all-cause mortality compared kidney cancer survivors to the general population at 6 months, 1 year, and 5 years after the index date.

	6 months after the index date					1 year after the index date					5 years after the index date				
	KCa survivors		Non-KCa individuals		Adjusted HR (95% CI)^a^	KCa survivors		Non-KCa individuals		Adjusted HR (95% CI)^a^	KCa survivors		Non-KCa individuals		Adjusted HR (95% CI)^a^
	N=20,093		N=129,139			N=20,093		N=129,139			N=20,093		N=129,139		
	N (%)	IR	N (%)	IR		N (%)	IR	N (%)	IR		N (%)	IR	N (%)	IR	
**CVD** ^b^	264 (1.31)	26.74	600 (0.46)	9.32	2.70 (2.31-3.15)	358 (1.78)	18.36	1,236 (0.96)	9.64	1.77 (1.56-2.00)	993 (4.94)	10.81	5,578 (4.32)	8.97	1.10 (1.03-1.18)
Myocardial infarction	14 (0.07)	1.41	61 (0.05)	0.95	1.40 (0.77-2.53)	20 (0.10)	1.01	126 (0.10)	0.98	0.96 (0.60-1.55)	104 (0.52)	1.10	624 (0.48)	0.98	0.99 (0.80-1.23)
Ischemic stroke/TIA	28 (0.14)	2.81	209 (0.16)	3.24	0.85 (0.57-1.29)	50 (0.25)	2.54	424 (0.33)	3.30	0.77 (0.57-1.04)	225 (1.12)	2.40	1,964 (1.52)	3.11	0.74 (0.64-0.85)
Hemorrhagic stroke	12 (0.06)	1.21	52 (0.04)	0.81	1.17 (0.62-2.21)	20 (0.10)	1.01	103 (0.08)	0.80	1.10 (0.68-1.80)	74 (0.37)	0.79	470 (0.36)	0.74	0.99 (0.77-1.26)
Atrial fibrillation	90 (0.45)	9.07	166 (0.13)	2.58	3.53 (2.68-4.64)	115 (0.57)	5.86	390 (0.30)	3.03	1.78 (1.43-2.22)	296 (1.47)	3.16	1,823 (1.41)	2.89	1.00 (0.88-1.13)
Heart failure	7 (0.03)	0.70	19 (0.01)	0.29	2.90 (1.16-7.27)	10 (0.05)	0.51	49 (0.04)	0.38	1.34 (0.67-2.69)	39 (0.19)	0.41	262 (0.20)	0.41	0.86 (0.61-1.22)
Peripheral arterial occlusion	32 (0.16)	3.22	72 (0.06)	1.12	2.22 (1.40-3.50)	42 (0.21)	2.13	142 (0.11)	1.10	1.51 (1.05-2.18)	121 (0.60)	1.29	589 (0.46)	0.93	1.10 (0.90-1.34)
Venous thromboembolism	89 (0.44)	8.96	47 (0.04)	0.73	11.54 (8.02-16.61)	114 (0.57)	5.80	94 (0.07)	0.73	7.55 (5.71-9.99)	241 (1.20)	2.57	511 (0.40)	0.81	3.05 (2.59-3.59)
**All-cause mortality^c^**	378 (1.88)	37.97	286 (0.22)	4.43	9.17 (7.75-10.86)	720 (3.58)	36.51	631 (0.49)	4.90	8.28 (7.37-9.32)	1,944 (9.68)	20.60	4,436 (3.44)	6.98	3.27 (3.08-3.47)

CI, confidence interval; CVD, cardiovascular disease; HR, hazard ratio; IR, incidence rate per 1,000 person-years; KCa, kidney cancer; TIA, transient ischemic attack.

^a^The models were adjusted for sociodemographic variables, comorbidities, medications, and lifestyle factors.

^b^For CVD risk, the Fine and Gray competing risk models were employed with the competing risk of death.

^c^For the risk of all-cause mortality, the Cox proportional hazards regression model was used.

Findings from the additional analyses indicate that the highest increased risk of CVD was observed at 3 months after KCa diagnosis (HR=4.24, 95% CI: 3.49-5.16), and the elevated CVD risk remained throughout the entire follow-up period after the index date (HR=1.05, 95% CI: 1.01-1.10), despite the risk weakening over time (**Supplementary Figure S2** and **Supplementary Table S4**).

### Secondary outcomes

At 6-month follow-up, KCa survivors had an increased risk of AF (HR=3.53, 95% CI: 2.68-4.64), heart failure (HR=2.90, 95% CI: 1.16-7.27), PAO (HR=2.22, 95% CI: 1.40-3.50), and VTE (HR=11.54, 95% CI: 8.02-16.61). After 1 year follow-up, KCa survivors were associated with significantly increased risks of AF (HR=1.78, 95% CI: 1.43-2.22), PAO (HR=1.51, 95% CI: 1.05-2.18), and VTE (HR=7.55, 95% CI: 5.71-9.99) compared to the comparison group. The increased risk of AF was shown up to 2-year follow-up (HR=1.29, 95% CI: 1.08-1.53), and the elevated risk of VTE remained after 5 years following the index date (HR=3.05, 95% CI: 2.59-3.59). Compared to general population, KCa survivors had 3.3-9.2 times increased risks of all-cause death over 5-year follow-up period.

### Subgroup and sensitivity analyses

**Figure 3:**
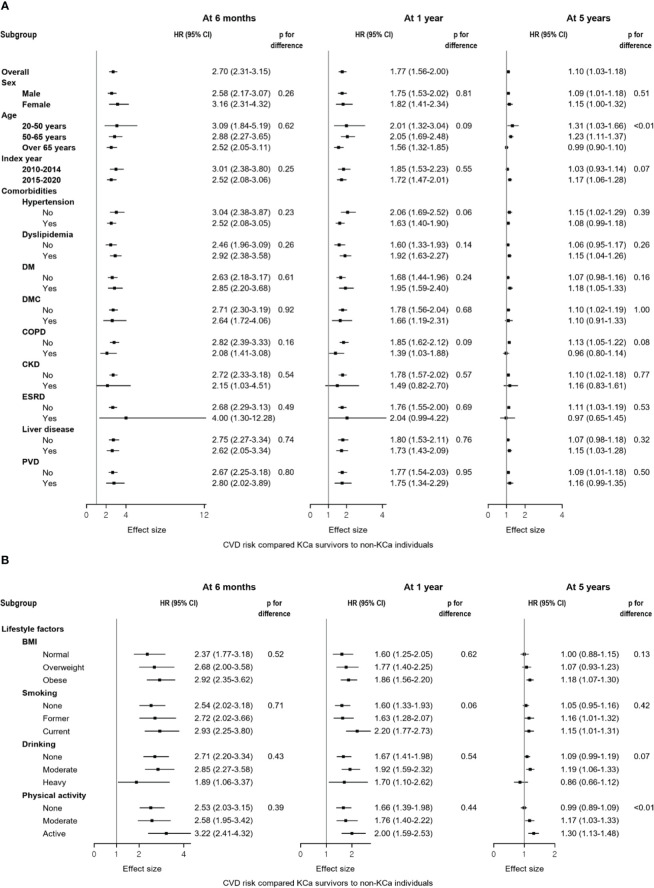
Forest plots of the subgroup analyses compared kidney cancer survivors to the general population at 6 months, 1 year, and 5 years after a cancer diagnosis. **(A)** Results from the subgroup analyses according to sex, age, index year, and comorbidities. **(B)** Results from the subgroup analyses according to lifestyle factors. Compared to non-KCa individuals, a higher risk of CVD was generally observed in KCa survivors across subgroups: **(A)** sex, age, index year, and comorbidities; **(B)** lifestyle factors. At 5 years after a cancer diagnosis, different results were observed within subgroups of age and physical activity. BMI, body mass index; CI, confidence interval; CKD, chronic kidney disease, COPD, chronic obstructive pulmonary disease, CVD, cardiovascular disease; DM, diabetes mellitus without complications; DMC, diabetes mellitus with complications; ESRD, end-stage renal disease; HR, hazard ratio; KCa, kidney cancer; PVD, peripheral vascular disease.

Results from the subgroup analyses generally align with the main findings (**Figures 3A, B**). For CVD risk at 5-year post cancer diagnosis, younger KCa patients were associated with a higher risk of CVD compared to the general population (age<50 year: HR=1.31, 95% CI: 1.03-1.66), while a null association was observed in those older than 65 years. Also, among individuals who were physically active, KCa survivors had an increased risk of CVD compared to the general population (HR=1.30, 95% CI: 1.13-1.48), whereas this association disappeared among those who were not physically active. Findings from the sensitivity analyses support the main findings (**Table 3**).

**Table 3:** Results from sensitivity analyses for the primary outcome compared KCa survivors to the general population at 6 months, 1 year, and 5 years after the index date.

	KCa survivors		Non-KCa individuals		Adjusted HR (95% CI)^a^
	N	Event, N (%)	N	Event, N (%)	
6 months after the index date					
Main analysis	20,093	264 (1.31)	129,139	600 (0.46)	2.70 (2.31-3.15)
Restriction of cancer survivors living longer than 2 years	17,290	191 (1.1)	117,509	508 (0.43)	2.46 (2.06-2.93)
Restriction of cancer survivors living longer than 5 years	10,841	109 (1.01)	75,747	286 (0.38)	2.61 (2.08-3.29)
Restriction of patients aged over 40 years	18,484	258 (1.40)	121,061	596 (0.49)	2.67 (2.29-3.12)
Applied IPTW method	19,865	261 (1.31)	129,228	606 (0.47)	2.72 (2.35-3.15)
Repeated analysis in the total population	32,954	463 (1.40)	238,224	1,296 (0.54)	2.53 (2.26-2.83)
1 year after the index date					
Main analysis	20,093	358 (1.78)	129,139	1,236 (0.96)	1.77 (1.56-2.00)
Restriction of cancer survivors living longer than 2 years	17,290	264 (1.53)	117,509	1,063 (0.88)	1.66 (1.44-1.91)
Restriction of cancer survivors living longer than 5 years	10,841	148 (1.37)	75,747	611 (0.81)	1.63 (1.35-1.96)
Restriction of patients aged over 40 years	18,484	350 (1.89)	121,061	1,228 (1.01)	1.75 (1.54-1.98)
Applied IPTW method	19,865	356 (1.79)	129,228	1,249 (0.97)	1.81 (1.61-2.04)
Repeated analysis in the total population	32,954	653 (1.98)	238,224	2,668 (1.12)	1.74 (1.59-1.90)
5 years after the index date					
Main analysis	20,093	993 (4.94)	129,139	5,578 (4.32)	1.10 (1.03-1.18)
Restriction of cancer survivors living longer than 2 years	17,290	877 (5.07)	117,509	5,265 (4.48)	1.11 (1.03-1.20)
Restriction of cancer survivors living longer than 5 years	10,841	541 (4.99)	75,747	3,479 (4.59)	1.07 (1.00-1.17)
Restriction of patients aged over 40 years	18,484	979 (5.30)	121,061	5,535 (4.57)	1.10 (1.03-1.18)
Applied IPTW method	19,865	970 (4.88)	129,228	5,621 (4.35)	1.10 (1.04-1.18)
Repeated analysis in the total population	32,954	1,698 (5.15)	238,224	10,872 (4.56)	1.11 (1.06-1.18)

CI, confidence interval; HR, hazard ratio; IPTW, stabilized inverse probability of treatment weighting methods; inverse KCa, kidney cancer.

^a^The models were adjusted for sociodemographic variables, comorbidities, medications, and lifestyle factors.

## Discussion

This nationwide, large population-based cohort study is the first, to our knowledge, to investigate the short- and long-term risk of CVD among KCa patients compared to the general population. We found that KCa survivors had approximately a 2.8-fold increased risk of CVD compared to the general population after 6 months following cancer diagnosis. Importantly, this heightened risk persisted up to five years post-diagnosis, while attenuated. Among the individual components of CVD, VTE and AF were the primary contributors of long-term CVD risk. Consistent findings according to various patient characteristics and from different sensitivity analyses support the main findings.

Although limited evidence exists regarding the risk of CVD among KCa survivors, previous studies, which did not primarily focus on KCa survivors as the primary study group nor cover the entire national population of Korea or other countries, have supported our findings. In one study conducted in Netherlands, an approximately two-fold higher risk of CVD was observed after 1-year follow-up in KCa survivors compared to non-KCa individuals, where CVD was defined as a composite of myocardial infarction, ischemic stroke, and PAO (21). However, they did not assess a long-term risk and did not account for smoking, which is a strong confounder of both KCa and CVD (32, 33). Another study also showed that survivors from combined bladder or kidney cancers were associated with a 7.3-times higher risk of 10-year atherosclerotic CVD risk score estimates compared to the general population (34). Hospitalized KCa survivors had a two-fold elevated risk of VTE compared to individuals without KCa (35). Additionally, a previous study conducted in Korea found that KCa survivors experienced a 2-times higher risk of AF after 3 months and 1 year following a cancer diagnosis, and this increased risk persisted at 1.4-times higher after 5 years (22). The AF risk at 5 years was found to be stronger than the risk estimate observed in our study. This difference may be attributed to the inclusion of patients with a history of ischemic heart disease, a well-known risk factor for AF (36), in their study, whereas our study excluded these individuals. Furthermore, existing evidence also support our findings, showing that cancer survivors were associated with an increased risk of CVD, especially AF, heart failure, or VTE, compared to the general population (37–39). Contrary to these previous studies, our study uniquely investigated the short- and long-term risk of CVD, by focusing on KCa survivors as the primary study population on a nationwide scale. Our data demonstrates the higher risk of CVD after KCa diagnosis and emphasizes the importance of prevention and management for CVD in addition to optimal cancer care among KCa survivors.

The current study included sufficient number of KCa survivors, and this allowed us to specifically explore which KCa survivors face a higher risk of CVD. The elevated risk of CVD was generally observed up to 5 years regardless of traditional risk factors for CVD, such as male sex, the presence of comorbidities, or unhealthy lifestyles (32). Interestingly, individuals diagnosed with KCa at age under 65 had a higher risk of CVD after 5-year follow-up compared to the general population, but this elevated risk disappeared among those older than 65. This suggests that KCa diagnosis has a more pronounced impact on an increasing long-term CVD risk in younger survivors versus older survivors. This may be attributed to older age, a well-known independent risk factors for CVD (32), which may play a more significant role in CVD risk than KCa diagnosis (5). Older age affects both individuals with and without KCa similarly, resulting in a similar CVD risk. Similarly, KCa diagnosis was more influential to individuals who were more physically active compared to those who were not. These findings suggest that, despite individuals being younger or having healthy lifestyles, KCa may increase the long-term risk of CVD. It is important to address these concerns, ensuring they are managed similarly to those of older or those with less healthy lifestyles.

Until now, CVD risk among KCa survivors has been overlooked compared to survivors from other cancer sites. Given an increasing trend of CVD death and a decreasing trend of cancer death among KCa survivors (4), CVD prevention and management among KCa survivors is crucial. Our findings emphasize the significance of developing evidence-based guidance for CVD care throughout cancer survivorship in order to improve overall survival and quality of life. Specifically, a multidisciplinary approach is critical in defining the best strategies for the prevention, early detection, and management of CVD in cancer survivors (17, 18). This approach will help determine when, how, and who should be involved in cardio-oncology care.

Beyond our findings, it is noteworthy that current therapies, such as immunotherapy or tyrosine kinase inhibitors, have dramatically improved cancer prognosis (3, 40). However, they have also led to unexpected CVD complications including cardiac failure, cardiomyopathy, arrhythmia, or thrombotic events (8, 9, 40, 41). These complications can range from asymptomatic and transient to clinically significant and long-term cardiac events. It is important to weigh potential CVD damage against the benefits of cancer therapy. Further research with detailed information on cancer treatments is warranted to expand the current findings.

Our study has several strengths. We used the nationwide, large population-based cohort, primarily focusing on a substantial number of KCa survivors. We ensured rigorous ascertainment of KCa diagnosis and CVD occurrences. Our long-term, rich data on clinical characteristics and lifestyle factors allowed for a comprehensive investigation of various CVD, minimizing potential bias. Several limitations also should be noted. First, there is a lack of information on tumor characteristics and cancer treatments, which could affect CVD risk (8, 9, 40, 41). However, considering that the vast majority (80%) of KCa patients receive nephrectomy and less than 10% receive systemic treatments or radiation therapy (4), the elevated CVD risk among KCa survivors in our study may be limitedly influenced by different treatment types. Second, due to the nature of retrospective study using recorded health insurance and survey data, residual confounding can exist, despite regression models adjusted for potential strong confounders, and robust findings from subgroup and sensitivity analyses. Also, we were unable to investigate whether CVD risk factors, including blood pressure, glucose, or lipid levels, were controlled and their impacts. Lastly, although our study cohort was drawn from the entire Korean population, thus representing the East Asian population, it is important to note that the generalizability of our findings may be limited when applied to other racial and ethnic groups. Compared to White population, Asians showed a 22% decreased risk of CVD mortality, whereas Black people showed a 37% increased risk among T1/T2 KCa survivors (5). Additional investigations are necessary to explore potential racial/ethnic disparities in CVD risk among KCa survivors.

## Conclusion

We demonstrated that KCa survivors were associated with an increased risk of CVD up to 5 years after cancer diagnosis compared to the general population. Our findings emphasize the importance of prevention and management for CVD among KCa survivors, while simultaneously providing optimal cancer care.

## Data availability statement

The data analyzed in this study is subject to the following licenses/restrictions: The study used the Korean Nationwide Health Insurance Database. The authors cannot legally distribute these data, but details on data information can be found here: https://nhiss.nhis.or.kr/bd/ab/bdaba012eng.do. Requests to access these datasets should be directed to MJ, mjjung@stanford.edu.

## Author contributions

MJ: Writing – review & editing, Writing – original draft, Validation, Project administration, Methodology, Investigation, Formal analysis, Conceptualization. EC: Writing – review & editing, Visualization, Investigation, Formal analysis, Data curation. SLi: Writing – review & editing, Methodology. ZD: Writing – review & editing. JL: Writing – review & editing. ML: Writing – review & editing. SB: Writing – review & editing. SLee: Resources, Writing – review & editing. MEL: Writing – review & editing, Supervision, Methodology, Investigation, Conceptualization. BIC: Methodology, Writing – review & editing, Supervision, Investigation, Conceptualization.
