# Supplementary material for: Increased risk of cardiovascular disease among kidney cancer survivors: a nationwide population-based cohort study
Source: Front Oncol. 2024 Jul 12;14:1420333. doi: 10.3389/fonc.2024.1420333 (PMC11272517; doi:10.3389/fonc.2024.1420333)
Supplement: Supplementary file 1 [file DataSheet_1.docx]

List of supplemental materials

Supplemental Table 1. ICD-10 codes which were used in this study

Supplemental Table 2. Index year distribution of the study population

Supplemental Table 3. Baseline characteristics of the total population including both individuals who took and did not take the national medical checkup survey

Supplemental Table 4. Among the study population, risks of the study outcomes compared kidney cancer survivors to the general population

Supplemental Figure 1. Graphical representation of study design

Supplemental Figure 2. Cumulative incidence curve of cardiovascular disease among kidney cancer survivors compared to the general population

Supplemental Table 1. ICD-10 codes which were used in this study

| ICD-10-CM Codes | |
| --- | --- |
| Kidney cancer | C64.x |
| Atrial fibrillation | I48.x |
| Hypertension | I10.x–I15.x |
| Dyslipidemia | E78.x |
| Chronic obstructive pulmonary disease | J41.x-J44.x |
| Renal failure, dialysis, or renal transplantation | I12.0, I13.11, N18.5, N18.6, N19.x, Z49.0, Z94.0, Z99.2 |
| Ischemic stroke/transient ischemic attack | I63.x, G45.x |
| Hemorrhagic stroke | I60.x-I62.x |
| Peripheral arterial occlusion | I74.x |
| Venous thromboembolism | I80.1-I80.3, I26.x |
| Charlson Comorbidity Index (CCI) | |
| Weight=1 | |
| Cerebrovascular disease | G45.x, G46.x, H34.0, I60.x-I69.x |
| Congestive heart failure | I50.x |
| Chronic pulmonary disease | I27.8, I27.9, J40.x–J47.x, J60.x–J67.x, J68.4, J70.1, J70.3 |
| Dementia | F01.x–F03.x, G30.x–G32.x |
| Diabetes without chronic complication | E10.0, E10.1, E10.6, E10.8, E10.9, E11.0, E11.1, E11.6, E11.8, E11.9, E13.0, E13.1, E13.6, E13.8, E13.9 |
| Mild liver disease | B18.x, K70.0-K70.3, K70.9, K71.3-K71.5, K71.7, K73.x, K74.x, K76.0, K76.2-K76.4, K76.8, K76.9, Z94.4 |
| Mild or moderate renal disease | I12.9, I13.0, I13.10, N03.x, N05.x, N18.1, N18.2, N18.3, N18.4, N18.9, Z94.0 |
| Myocardial infarction | I21.x, I22.x, I25.2 |
| Peripheral vascular disease | I70.x, I71.x, I73.1, I73.8, I73.9, I77.1, I79.0, I79.2, K55.1, K55.8, K55.9, Z95.8, Z95.9 |
| Peptic ulcer disease | K25.x–K28.x, |
| Rheumatologic disease | M05.x, M06.x, M32.x–M34.x M31.5M35.1, M35.3, M36.0 |
| **Weight=2** | |
| Diabetes with chronic complication | E10.2–E10.5, E11.2–E11.5, E13.2–E13.5, E14.x |
| Hemiplegia or paraplegia | G04.1, G11.4, G80.1, G80.2, G81.x, G82.x, G83.x |
| Any malignancy, including leukemia and lymphoma | C00.x–C26.x, C30.x–C34.x, C37.x–C41.x, C43.x, C45.x–C58.x, C60.x–C76.x, C81.x–C85.x, C88.x, C90.x–C97.x, |
| **Weight=3** | |
| Moderate or severe liver disease | I85.0x, I86.4, K70.4x, K71.1x, K72.1x, K72.9x, K76.5, K76.6, K76.7 |
| Severe renal disease | I12.0, I13.x, N03.2-N03.7, N05.2-N05.7, N18.5, N18.6, N19.x, N25.0, Z49.0-Z49.2, Z94.0, Z99.2 |
| **Weight=6** | |
| HIV/AIDS | B20.x |
| Metastatic solid tumor | C77.x–C80.0, C80.2 |
| Abbreviations. HIV/AIDS, human immunodeficiency virus infection and acquired immunodeficiency syndrome; ICD, International Classification of Diseases | |

Supplemental Table 2. Index year distribution of the study population

|  | Overall | | KCa survivors | | Non-KCa individuals | |
| --- | --- | --- | --- | --- | --- | --- |
|  | N=149,232 | | N=20,093 | | N=129,139 | |
|  | N | (%) | N | (%) | N | (%) |
| Index year (year of kidney cancer diagnosis) |  |  |  |  |  |  |
| 2010 | 8,673 | (5.81) | 1,168 | (5.81) | 7,505 | (5.81) |
| 2011 | 12,146 | (8.14) | 1,593 | (7.93) | 10,553 | (8.17) |
| 2012 | 12,926 | (8.66) | 1,706 | (8.49) | 11,220 | (8.69) |
| 2013 | 13,429 | (9.00) | 1,783 | (8.87) | 11,646 | (9.02) |
| 2014 | 13,765 | (9.22) | 1,922 | (9.57) | 11,843 | (9.17) |
| 2015 | 14,084 | (9.44) | 1,907 | (9.49) | 12,177 | (9.43) |
| 2016 | 16,240 | (10.88) | 2,173 | (10.81) | 14,067 | (10.89) |
| 2017 | 17,504 | (11.73) | 2,341 | (11.65) | 15,163 | (11.74) |
| 2018 | 15,071 | (10.10) | 1,956 | (9.73) | 13,115 | (10.16) |
| 2019 | 13,460 | (9.02) | 1,838 | (9.15) | 11,622 | (9.00) |
| 2020 | 11,934 | (8.00) | 1,706 | (8.49) | 10,228 | (7.92) |
| Abbreviations. KCa, kidney cancer | | | | | | |

Supplemental Table 3. Baseline characteristics of the total population including both individuals who took and did not take the national medical checkup survey

|  | Overall | | KCa survivors | | Non-KCa individuals | |
| --- | --- | --- | --- | --- | --- | --- |
|  | N=271,178 | | N=32,954 | | N=238,224 | |
|  | N | (%) | N | (%) | N | (%) |
| Sex |  |  |  |  |  |  |
| Male | 185,308 | 68.33 | 22,539 | 68.40 | 162,769 | 68.33 |
| Female | 85,870 | 31.67 | 10,415 | 31.60 | 75,455 | 31.67 |
| Age (Median (IQR)) | 59 | (50-68) | 57 | (48-66) | 59 | (50-69) |
| Under 50 years | 66,092 | 24.37 | 9,224 | 27.99 | 56,868 | 23.87 |
| 50-65 years | 112,247 | 41.39 | 14,250 | 43.24 | 97,997 | 41.14 |
| Over 65 years | 92,839 | 34.24 | 9,480 | 28.77 | 83,359 | 34.99 |
| Income level^a^ |  |  |  |  |  |  |
| Low | 65,392 | 24.11 | 7,187 | 21.81 | 58,205 | 24.43 |
| Middle | 75,338 | 27.78 | 8,792 | 26.68 | 66,546 | 27.93 |
| High | 125,352 | 46.22 | 16,389 | 49.73 | 108,963 | 45.74 |
| Missing | 5,096 | 1.88 | 586 | 1.78 | 4,510 | 1.89 |
| Number of visits to medical institutions^b^ (Median (IQR)) | 13 | (6-23) | 17 | (9-28) | 12 | (5-22) |
| Frequent visitors | 66,075 | 24.37 | 11,110 | 33.71 | 54,965 | 23.07 |
| Comorbidity |  |  |  |  |  |  |
| Hypertension | 99,860 | 36.82 | 16,005 | 48.57 | 83,855 | 35.20 |
| Dyslipidemia | 88,483 | 32.63 | 15,424 | 46.80 | 73,059 | 30.67 |
| Diabetes without complications | 48,122 | 17.75 | 8,668 | 26.30 | 39,454 | 16.56 |
| Diabetes with complications | 16,190 | 5.97 | 2,973 | 9.02 | 13,217 | 5.55 |
| COPD | 21,953 | 8.10 | 4,137 | 12.55 | 17,816 | 7.48 |
| Chronic kidney disease | 3,284 | 1.21 | 1,327 | 4.03 | 1,957 | 0.82 |
| End-stage renal disease | 2,214 | 0.82 | 1,044 | 3.17 | 1,170 | 0.49 |
| Mild to moderate liver disease | 61,056 | 22.52 | 13,557 | 41.14 | 47,499 | 19.94 |
| Severe liver disease | 799 | 0.29 | 177 | 0.54 | 622 | 0.26 |
| Peripheral vascular disease | 28,931 | 10.67 | 4,009 | 12.17 | 24,922 | 10.46 |
| CCI scores (Median (IQR)) | 1 | (0-2) | 1 | (1-3) | 1 | (0-2) |
| 0 | 112,425 | 41.46 | 7,983 | 24.22 | 104,442 | 43.84 |
| 1 | 74,224 | 27.37 | 9,178 | 27.85 | 65,046 | 27.30 |
| 2 | 42,022 | 15.50 | 6,941 | 21.06 | 35,081 | 14.73 |
| Over 3 | 42,507 | 15.67 | 8,852 | 26.86 | 33,655 | 14.13 |
| Medication |  |  |  |  |  |  |
| ACEI/ARB | 63,659 | 23.47 | 10,423 | 31.63 | 53,236 | 22.35 |
| Calcium channel blockers | 59,046 | 21.77 | 9,295 | 28.21 | 49,751 | 20.88 |
| Diuretics | 30,677 | 11.31 | 4,895 | 14.85 | 25,782 | 10.82 |
| Statin | 48,860 | 18.02 | 7,332 | 22.25 | 41,528 | 17.43 |
| Metformin | 28,733 | 10.60 | 4,061 | 12.32 | 24,672 | 10.36 |
| Abbreviations. ACEI/ARB, angiotensin converting enzyme inhibitors/angiotensin receptor blocker; COPD, chronic obstructive pulmonary disease; IQR, interquartile range; KCa, kidney cancer; SMD, absolute standardized mean difference  ^a^ Income level was identified based on the third quartile.  ^b^ Patients who visited medical institutions more than 24 times during one year prior to the index date were defined as “frequent visitors”. | | | | | | |

Supplemental Table 4. Among the study population, risks of the study outcomes compared kidney cancer survivors to the general population

|  | KCa survivors | Non-KCa individuals | Crude HR (95% CI) | Adjusted HR (95% CI)^a^ |
| --- | --- | --- | --- | --- |
|  | N=20,093 | N=129,139 |  |  |
|  | N (%) | N (%) |  |  |
| **3 months after cancer diagnosis** |  |  |  |  |
| CVD^b^ | 200 (1.00) | 284 (0.22) | 4.55 (3.79-5.45) | 4.24 (3.49-5.16) |
| Myocardial infarction | 9 (0.04) | 32 (0.02) | 1.81 (0.86-3.79) | 1.51 (0.71-3.23) |
| Ischemic stroke/TIA | 15 (0.07) | 99 (0.08) | 0.97 (0.57-1.68) | 0.99 (0.56-1.73) |
| Hemorrhagic stroke | 5 (0.02) | 26 (0.02) | 1.24 (0.47-3.22) | 1.03 (0.38-2.80) |
| Atrial fibrillation | 75 (0.37) | 77 (0.06) | 6.27 (4.56-8.62) | 6.30 (4.47-8.89) |
| Heart failure | 6 (0.03) | 8 (0.01) | 4.82 (1.67-13.90) | 6.36 (2.20-18.42) |
| Peripheral arterial occlusion | 28 (0.14) | 33 (0.03) | 5.46 (3.30-9.03) | 3.83 (2.15-6.83) |
| Venous thromboembolism | 68 (0.34) | 17 (0.01) | 25.75 (15.13-43.81) | 23.40 (13.38-40.92) |
| All-cause mortality^c^ | 176 (0.88) | 131 (0.10) | 8.67 (6.91-10.87) | 9.02 (7.05-11.54) |
| **6 months after cancer diagnosis** |  |  |  |  |
| CVD^b^ | 264 (1.31) | 600 (0.46) | 2.85 (2.46-3.29) | 2.70 (2.31-3.15) |
| Myocardial infarction | 14 (0.07) | 61 (0.05) | 1.48 (0.83-2.64) | 1.40 (0.77-2.53) |
| Ischemic stroke/TIA | 28 (0.14) | 209 (0.16) | 0.86 (0.58-1.28) | 0.85 (0.57-1.29) |
| Hemorrhagic stroke | 12 (0.06) | 52 (0.04) | 1.48 (0.79-2.78) | 1.17 (0.62-2.21) |
| Atrial fibrillation | 90 (0.45) | 166 (0.13) | 3.49 (2.70-4.52) | 3.53 (2.68-4.64) |
| Heart failure | 7 (0.03) | 19 (0.01) | 2.37 (1.00-5.63) | 2.90 (1.16-7.27) |
| Peripheral arterial occlusion | 32 (0.16) | 72 (0.06) | 2.86 (1.89-4.34) | 2.22 (1.40-3.50) |
| Venous thromboembolism | 89 (0.44) | 47 (0.04) | 12.20 (8.57-17.37) | 11.54 (8.02-16.61) |
| All-cause mortality^c^ | 378 (1.88) | 286 (0.22) | 8.57 (7.35-9.99) | 9.17 (7.75-10.86) |
| **1 year after cancer diagnosis** |  |  |  |  |
| CVD^b^ | 358 (1.78) | 1,236 (0.96) | 1.88 (1.67-2.11) | 1.77 (1.56-2.00) |
| Myocardial infarction | 20 (0.10) | 126 (0.10) | 1.02 (0.64-1.64) | 0.96 (0.60-1.55) |
| Ischemic stroke/TIA | 50 (0.25) | 424 (0.33) | 0.76 (0.57-1.02) | 0.77 (0.57-1.04) |
| Hemorrhagic stroke | 20 (0.10) | 103 (0.08) | 1.25 (0.77-2.02) | 1.10 (0.68-1.80) |
| Atrial fibrillation | 115 (0.57) | 390 (0.30) | 1.90 (1.54-2.34) | 1.78 (1.43-2.22) |
| Heart failure | 10 (0.05) | 49 (0.04) | 1.31 (0.66-2.59) | 1.34 (0.67-2.69) |
| Peripheral arterial occlusion | 42 (0.21) | 142 (0.11) | 1.90 (1.35-2.69) | 1.51 (1.05-2.18) |
| Venous thromboembolism | 114 (0.57) | 94 (0.07) | 7.82 (5.95-10.27) | 7.55 (5.71-9.99) |
| All-cause mortality^c^ | 720 (3.58) | 631 (0.49) | 7.46 (6.70-8.30) | 8.28 (7.37-9.32) |
| **2 years after cancer diagnosis** |  |  |  |  |
| CVD^b^ | 552 (2.75) | 2,480 (1.92) | 1.44 (1.32-1.58) | 1.37 (1.25-1.51) |
| Myocardial infarction | 39 (0.19) | 285 (0.22) | 0.88 (0.63-1.23) | 0.82 (0.58-1.16) |
| Ischemic stroke/TIA | 102 (0.51) | 799 (0.62) | 0.82 (0.67-1.01) | 0.82 (0.66-1.01) |
| Hemorrhagic stroke | 34 (0.17) | 201 (0.16) | 1.09 (0.76-1.56) | 0.99 (0.69-1.44) |
| Atrial fibrillation | 168 (0.84) | 818 (0.63) | 1.32 (1.12-1.56) | 1.29 (1.08-1.53) |
| Heart failure | 14 (0.07) | 111 (0.09) | 0.81 (0.47-1.41) | 0.72 (0.41-1.29) |
| Peripheral arterial occlusion | 63 (0.31) | 283 (0.22) | 1.43 (1.09-1.88) | 1.14 (0.86-1.51) |
| Venous thromboembolism | 159 (0.79) | 208 (0.16) | 4.93 (4.01-6.06) | 5.03 (4.05-6.25) |
| All-cause mortality^c^ | 1,151 (5.73) | 1,475 (1.14) | 5.16 (4.77-5.57) | 5.73 (5.26-6.25) |
| **3 years after cancer diagnosis** |  |  |  |  |
| CVD^b^ | 724 (3.60) | 3,643 (2.82) | 1.29 (1.19-1.39) | 1.23 (1.13-1.33) |
| Myocardial infarction | 63 (0.31) | 422 (0.33) | 0.96 (0.74-1.25) | 0.87 (0.66-1.15) |
| Ischemic stroke/TIA | 145 (0.72) | 1,230 (0.95) | 0.76 (0.64-0.90) | 0.77 (0.64-0.91) |
| Hemorrhagic stroke | 50 (0.25) | 299 (0.23) | 1.07 (0.80-1.45) | 1.00 (0.73-1.35) |
| Atrial fibrillation | 213 (1.06) | 1,186 (0.92) | 1.16 (1.00-1.34) | 1.09 (0.94-1.27) |
| Heart failure | 21 (0.10) | 171 (0.13) | 0.79 (0.50-1.24) | 0.68 (0.43-1.08) |
| Peripheral arterial occlusion | 85 (0.42) | 392 (0.30) | 1.40 (1.10-1.76) | 1.15 (0.91-1.47) |
| Venous thromboembolism | 199 (0.99) | 310 (0.24) | 4.15 (3.47-4.95) | 4.15 (3.43-5.01) |
| All-cause mortality^c^ | 1,468 (7.31) | 2,434 (1.88) | 4.02 (3.77-4.29) | 4.48 (4.17-4.81) |
| **4 years after cancer diagnosis** |  |  |  |  |
| CVD^b^ | 863 (4.30) | 4,714 (3.65) | 1.19 (1.10-1.28) | 1.14 (1.06-1.23) |
| Myocardial infarction | 81 (0.40) | 534 (0.41) | 0.97 (0.77-1.23) | 0.90 (0.71-1.15) |
| Ischemic stroke/TIA | 176 (0.88) | 1,638 (1.27) | 0.69 (0.59-0.81) | 0.70 (0.60-0.82) |
| Hemorrhagic stroke | 64 (0.32) | 400 (0.31) | 1.03 (0.79-1.34) | 0.99 (0.75-1.30) |
| Atrial fibrillation | 253 (1.26) | 1,544 (1.20) | 1.06 (0.92-1.21) | 1.01 (0.88-1.15) |
| Heart failure | 32 (0.16) | 228 (0.18) | 0.90 (0.62-1.31) | 0.79 (0.54-1.15) |
| Peripheral arterial occlusion | 105 (0.52) | 493 (0.38) | 1.37 (1.11-1.69) | 1.13 (0.91-1.41) |
| Venous thromboembolism | 230 (1.14) | 406 (0.31) | 3.66 (3.11-4.30) | 3.73 (3.14-4.42) |
| All-cause mortality^c^ | 1,740 (8.66) | 3,447 (2.67) | 3.38 (3.19-3.59) | 3.76 (3.53-4.01) |
| **5 years after cancer diagnosis** |  |  |  |  |
| CVD^b^ | 993 (4.94) | 5,578 (4.32) | 1.15 (1.08-1.23) | 1.10 (1.03-1.18) |
| Myocardial infarction | 104 (0.52) | 624 (0.48) | 1.07 (0.87-1.32) | 0.99 (0.80-1.23) |
| Ischemic stroke/TIA | 225 (1.12) | 1,964 (1.52) | 0.74 (0.64-0.84) | 0.74 (0.64-0.85) |
| Hemorrhagic stroke | 74 (0.37) | 470 (0.36) | 1.01 (0.79-1.29) | 0.99 (0.77-1.26) |
| Atrial fibrillation | 296 (1.47) | 1,823 (1.41) | 1.05 (0.93-1.18) | 1.00 (0.88-1.13) |
| Heart failure | 39 (0.19) | 262 (0.20) | 0.96 (0.68-1.34) | 0.86 (0.61-1.22) |
| Peripheral arterial occlusion | 121 (0.60) | 589 (0.46) | 1.32 (1.09-1.61) | 1.10 (0.90-1.34) |
| Venous thromboembolism | 241 (1.20) | 511 (0.40) | 3.05 (2.62-3.55) | 3.05 (2.59-3.59) |
| All-cause mortality^c^ | 1,944 (9.68) | 4,436 (3.44) | 2.95 (2.80-3.11) | 3.27 (3.08-3.47) |
| Abbreviations. CI, confidence interval; CVD, cardiovascular disease; HR, hazard ratio; IR, incidence rate per 1,000 person-years; KCa, kidney cancer; TIA, transient ischemic attack  a The models were adjusted for sociodemographic variables, comorbidities, medications, and lifestyle factors.  b For CVD risk, the Fine and Gray competing risk models were employed with the competing risk of death.  c For the risk of all-cause mortality, the Cox proportional hazards regression model was used. | | | | |

Supplemental Figure 1. Graphical representation of study design


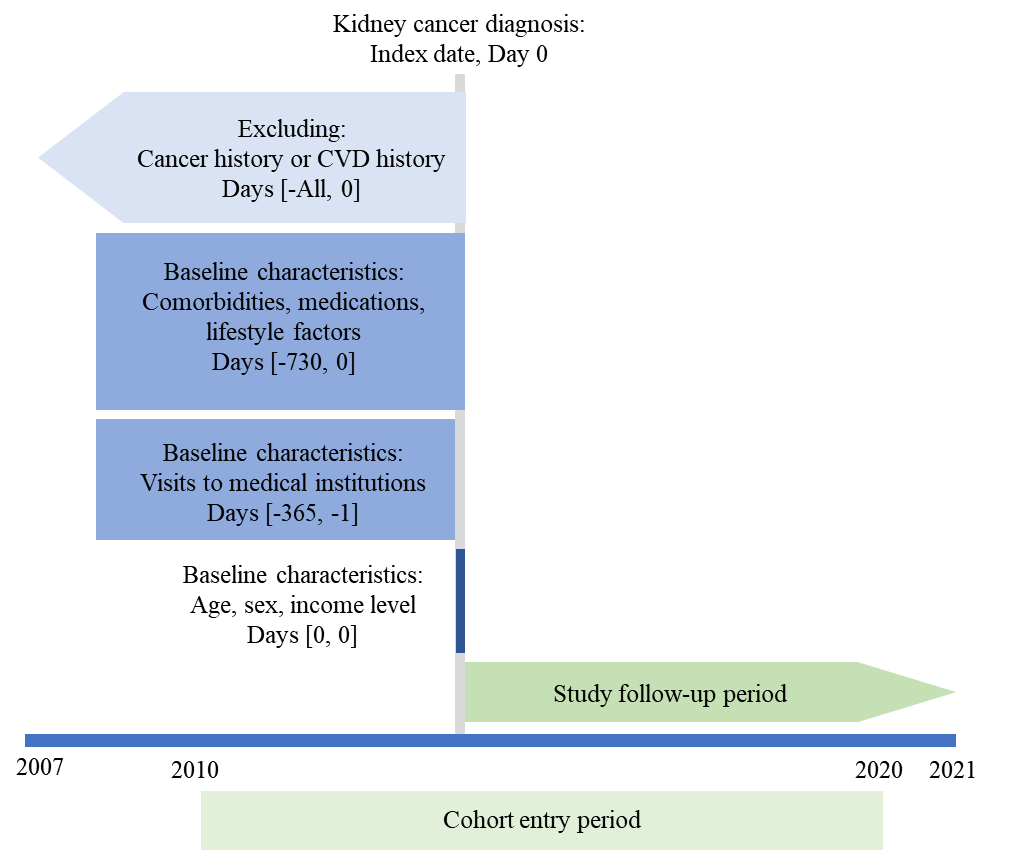


Supplementary Figure 1 Legend.

We included individuals between 2010 and 2020, which was defined as a cohort entry period. Baseline characteristics including comorbidities and medication use from the claims data and lifestyle factors from the medical checkup data were identified within 2 years prior to the index date. Each participant was followed from the index date to the occurrence of outcome, death, or end-date of the study period (December 31, 2021), whichever came first.

Abbreviations. CVD, cardiovascular disease

Supplemental Figure 2. Cumulative incidence curve of cardiovascular disease among kidney cancer survivors compared to the general population


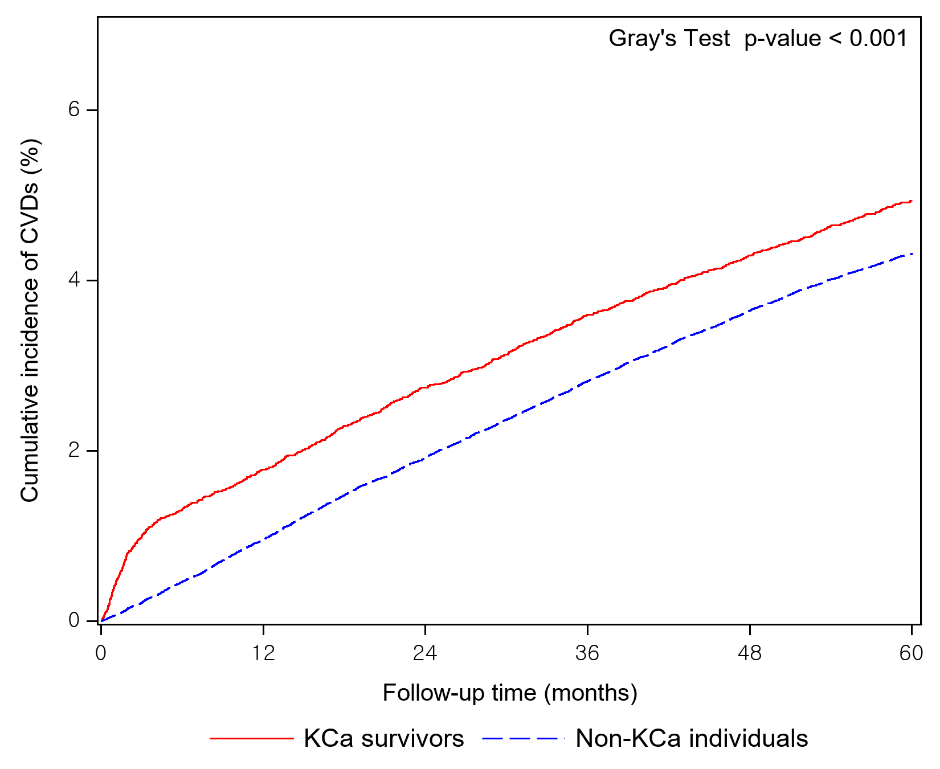


Supplementary Figure 2 Legend.

Increased risk of CVD was observed up to 5 years when compared KCa survivors to the general population.

Abbreviations. CVD, cardiovascular disease; KCa, kidney cancer
